# WHO five moments for medication safety among diabetic patients in Saudi Arabia

**DOI:** 10.1371/journal.pone.0346935

**Published:** 2026-04-10

**Authors:** Ayat ElZayat, Rawaa Jilani, Assel Krimly, Lamar Awan, Lamar Alsiamy, Nawal Baeshin

**Affiliations:** 1 Clinical Sciences Department- MBBS Program, Fakeeh College for Medical Sciences, Fakeeh Care Group, Jeddah, Saudi Arabia; 2 Bachelor of Medicine and Surgery- MBBS Program, Fakeeh College for Medical Sciences, Fakeeh Care Group, Jeddah, Saudi Arabia; Jazan Health Cluster, SAUDI ARABIA

## Abstract

**Background:**

Diabetic patients are particularly vulnerable to medication errors. Assessing adherence to the WHO Five Moments for Medication Safety among diabetic patients is essential for identifying gaps in practice and improving patient safety in this high-risk group. This is the first study to assess medication practices based on the WHO Five Moments among diabetic patients in Saudi Arabia.

**Methods:**

This cross-sectional study was conducted on 473 adult diabetic patients in Jeddah, Saudi Arabia. Patients completed a structured, self-administered questionnaire. The questionnaire comprised questions of the WHO Five Moments for Medication Safety. Content validity was confirmed through expert review, translation to Arabic followed forward–forward-backward procedure, and pre-testing confirmed face validity.

**Results:**

Diabetic patients generally exhibited the highest practice in moment two: taking medications (72%) and the lowest in moment five: stopping medications (54%), and 66% showed high overall medication safety practice. Multiple regression identified that following a regimen, using hypoglycaemic medication or insulin, and controlled HbA1c as positive predictors of total medication safety scores (P-value <0.05).

**Conclusion:**

Diabetic patients in Saudi Arabia generally exhibited overall a high adherence to the “Five Moments for Medication Safety,” with the highest practice in taking medications and the lowest in stopping them.

## Introduction

Patient safety has become a key component of the healthcare system because there is a lot of evidence that medical errors are common, and with an increasing prevalence [1]. A vital side of patient safety involves medication safety, as the harm due to medication errors is significant but preventable [2]. Medication errors can result in ineffective treatment, adverse drug reactions, increased hospitalisations and prolonged length of stay. In severe cases, these errors may lead to long-term complications or even death [3].

Medication errors can occur not only at the level of contacting the healthcare providers, but also extend to the patient level at the administration phase, especially when they are used at home [4]. Therefore, the patient plays a critical role in ensuring medication safety and preventing errors [5]. To draw attention to this role, the World Health Organisation (WHO) introduced the “Five Moments for Medication Safety,” a practical tool designed to ensure medication safety practices. These five moments identify critical points at which the patient should take action and be alert to prevent errors [6].

These five moments include safety practices, namely “starting a medication”, “taking my medication”, “adding a medication”, “reviewing my medication” and “stopping my medication”, as moments one to five, respectively [7]. They focus on engaging patients and caregivers in the medication process rather than passively depending on the healthcare providers [8]. This active role of patients can build patients’ self-confidence, prevent harm and ensure medication safety [9].

Patient engagement means that patients are involved in their own care and work with healthcare providers. Patients who are involved in their care are more likely to follow treatment plans and stick to safety practices [10]. In terms of medication safety, this engagement prevents errors by getting patients to understand, question, and handle their medications correctly [11]. Teaching and engaging patients, especially those with chronic diseases like diabetes, makes treatment safer and more effective [8,9].

Complex regimens for treatment, polypharmacy, and comorbidities make diabetic patients particularly vulnerable to medication errors [12]. As a lifelong disease, it relies profoundly on patients for proper management. Medication errors in such a population can lead to serious complications such as hypoglycemia, recurrent and/or serious infections, and hospitalization [13]. Assessing adherence to the WHO Five Moments among diabetic patients is therefore essential to identify gaps in practice, improve patient safety, and optimise treatment outcomes in this high-risk group.

To the best of our knowledge, this is the first study to assess how diabetic patients employ safe medication practices based on the WHO Five Moments in Saudi Arabia. This may provide a foundation for policymakers to develop and implement interventions to improve medication safety and minimize medical errors, especially among this vulnerable group.

## Methods

### Study design, setting, and participants

This cross-sectional study was conducted among diabetic patients in Jeddah, Saudi Arabia, between January 1 and June 30, 2025. Inclusion criteria were being adult patients (≥18 years) diagnosed with type I or type II diabetes mellitus, both sexes, currently taking at least one antidiabetic medication (oral hypoglycaemic agent and/or insulin), and receiving care in either private or public healthcare settings in Saudi Arabia. Exclusion criteria were those with gestational diabetes and diabetic patients with a medical or healthcare background, as prior professional knowledge may influence their medication safety practices.

### Sample size and design

The sample size was calculated using Epi Info (version 3.01; Centers for Disease Control and Prevention, USA) assuming a population size of 10,000, a 95% confidence level, and a 5% margin of error, based on the reported prevalence (20.2%) from a previous study conducted in Dammam, Saudi Arabia [14]. The minimum calculated sample size was 248 participants; however, the sample size was increased to compensate for possible non-response or incomplete data. Convenient sampling was used for recruiting participants by oncoming eligible diabetic patients attending outpatient clinics in both private and public healthcare settings in Jeddah, Saudi Arabia.

### Data collection tool and procedure

The questionnaire included the following three sections “S1 Appendix”:

First: questions related to the general characteristics of the students, including age, sex, marital status, highest educational level and occupation.
Second: questions related to clinical data, including type of diabetes, following a specific regimen (diet or exercise), taking oral hypoglycaemic medication, taking insulin injections, glycaemic control based on the latest HbA1c, having any Co-morbidities, and type of healthcare provider.
Third: questions related to the practice of medication safety were developed by adapting the WHO Five Moments for Medication Safety framework into 25 relevant items across five domains: “starting a medicine”, “taking my medicine”, “adding a medicine”, “reviewing my medicine”, and “stopping my medicine” [7]. Each domain has 5 items with a total of 25 items that were converted to questions with a 3-likert frequency scale answers as follows: Never –Sometimes –Always, with the corresponding scores of (0–2). For each moment, the score was calculated by summing the score for each of its five questions with the score of the moment range (0–10). The total score of medication safety practices was calculated by summing the scores for the five moments with score ranges (0–50). The higher scores denote better practice.

Due to the lack of a universal cut-off point for categorizing medication safety practices, this study used the approach followed by previous studies [15–17]. Participants’ responses were categorized into three categories based on percentile scores: 0–33% (scores 0–3) were classified as low practice, 34–66% (scores 4–6) as moderate practice, and 67–100% (scores 7–10) as high practice.

### Validity and reliability

The items of WHO Five Moments for Medication Safety were converted into relevant questions. Content validity of the developed questionnaire was assessed by an expert panel review. The panel included five experts in clinical pharmacy, patient safety, diabetes, and public health. Each expert independently assessed the questionnaire items for clarity and relevance, using an evaluation form. Their feedback was collected, and modifications were made to ensure that the instrument represents the content of medication safety practices.

The questionnaire was translated into Arabic following the forward–backward translation approach [18]. First, two independent experts, fluent in both English and Arabic, performed the forward translation from English to Arabic. The translations were merged into a single version after removing any discrepancies. Next, another two independent translators, who were blind to the original developed questionnaire, performed a backward translation from Arabic to English. The backward translation was compared with the original English version to check for its equivalence. Any inconsistencies were discussed and corrected until an agreement was reached.

The final Arabic version was pre-tested on a small group of diabetic patients as a pilot study for face validity to ensure clarity, comprehensibility, and cultural appropriateness before use in the study. Internal consistency reliability was calculated for the questions of each moment and the total questionnaire, where Cronbach’s Alpha >0.7 was considered acceptable, “Table 1”.

**Table 1:** Internal consistency reliability of the WHO medication safety questionnaire.

Scale	Cronbach’s Alpha
Moment one: starting a medicine	0.724
Moment two: taking my medicine	0.747
Moment three: adding a medicine	0.835
Moment four: reviewing my medicine	0.785
Moment five: stopping my medicine	0.809
Total scale	0.928

### Ethical considerations

The study was approved by the Institutional Review Board of Fakeeh College of Medical Sciences, Jeddah (No. 539/IRB/2023). After explaining the purpose of the study, ensuring confidentiality of data, and clarifying that participation was entirely voluntary, eligible participants were provided with a unique code to complete the questionnaire. They were informed that they could withdraw at any time without consequence, and all responses would remain anonymous, coded, and used solely for research purposes. The study utilized a written consent process at the beginning of the self-administered questionnaire. To be included, each participant was required to provide verbal consent. The estimated time to complete the questionnaire was about 10–12 minutes.

There were no rewards for taking part. Before distribution, the authors tested the electronic questionnaire’s technical functionality and usability. By choosing a Google Forms option, participants were notified that their response had already been sent in case they tried to submit it again, preventing duplicate submissions. Participants completed the questionnaire independently in a private setting after verifying eligibility. Assistance was provided if any participant needed to clarify questions without influencing their responses.

### Data management and analysis

Data were coded, entered, and managed using Microsoft Excel (2019 version). Data analyses were performed using IBM SPSS software (version 25). The final analysis only contained responses with no missing data. Descriptive statistics were presented by frequencies and percentages for qualitative data and mean ± SD and median for quantitative data. The Kolmogorov-Smirnov test was used to determine the normal distribution of data. The Mann-Whitney test was used to compare means between two groups, and the Kruskal-Wallis test to compare means among more than two groups. The multivariate regression analysis was used to assess the factors affecting scores of medication safety practices. *P-value* <0.05 was considered statistically significant based on the level of confidence of 95%. Tables and figures were used to present the data.

## Results

The study included a total of 473 diabetic patients with an average age of 46 ± 12 years and a range of 18–60 years; 56.4% were female, and the majority were married (66.6%). Most of the participants had completed college education (50.3%), and nearly half of the participants were employed (49.7%), “Table 2”.

**Table 2:** General characteristics of the studied participants (n = 473).

General characteristics		Frequency	Percentage
**Sex**	Male	206	43.6
	Female	267	56.4
**Marital status**	Single	107	22.6
	Divorced	32	6.8
	Widowed	19	4.0
	Married	315	66.6
**Highest educational level**	Primary school	11	2.3
	Preparatory school	15	3.2
	High school	70	14.8
	Diploma	62	13.1
	College	238	50.3
	Post-graduate studies	77	16.3
**Occupation**	Student	71	15.0
	Employee	235	49.7
	Retiree	167	35.3

About 57% of the participants were diagnosed with type II diabetes. Slightly more than half of the participants (56.9%) reported following a specific regimen, such as diet or exercise, and 28.8% were taking oral hypoglycaemic agents, whereas 150 (31.7%) were using insulin injections. The majority (71.2%) demonstrated good glycaemic control based on the latest HbA1c measurements. Co-morbidities were present among 53.1%, and about 43.1% of the participants were followed up in the private sector, “Table 3”.

**Table 3:** Clinical data of the studied participants (n = 473).

Clinical data		Frequency	Percentage
**Type of diabetes mellitus**	Type I	203	42.9
	Type II	270	57.1
**Following a specific regimen (diet or exercise)**	Yes	269	56.9
	No	204	43.1
**Taking oral hypoglycaemic medication**	Yes	337	71.2
	No	136	28.8
**Taking insulin injections**	Yes	150	31.7
	No	323	68.3
**Glycaemic control based on the latest HbA1c**	Controlled	337	71.2
	Not controlled	136	28.8
**Co-morbidities**	Present	222	53.1
	Absent	251	46.9
**Type of healthcare provider**	Private sector	204	43.1
	Public sector	171	36.2
	Both	98	20.7

**Fig 1:**
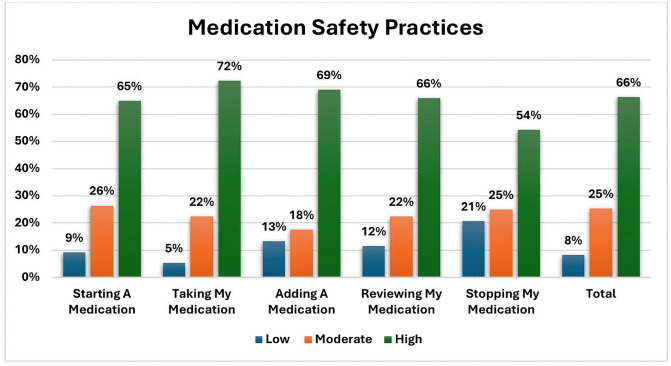
Medication safety practices among the studied participants (n = 473). Participants’ sex, marital status, and educational level were not significantly associated with scores across all medication safety practices (P > 0.05). However, students had higher scores in Moment Three (adding a medication), Moment Four (reviewing medications), and the total medication safety practices compared to employees and retired participants (P < 0.05) (Table 4).

The participants’ practices of the “Five Moments for Medication Safety” were generally high across all domains. The highest level of practice was observed in moment two: taking medications, with 72% demonstrating high practice, followed by moment three: adding a medication (69%), moment four: reviewing medications (66%), and moment one: starting a medication (65%). Stopping a medication, moment five, showed a slightly lower practice level at 54%. Overall, 66% of participants demonstrated high overall medication safety practices, “Fig 1”.

**Table 4:** Associations between general characteristics and medication safety practices among the studied participants (n = 473).

General characteristics		Starting a medication	Taking my medication	Adding a medication	Reviewing my medication	Stopping my medication	Total
**Sex**	Male	7.3 ± 3	8 ± 2	7.5 ± 3	7.3 ± 3	6.2 ± 3	36.3 ± 12
	Female	7.4 ± 3	8 ± 3	7.5 ± 3	7.4 ± 3	6.8 ± 3	37 ± 13
	*P-value*	*0.255*	*0.688*	*0.769*	*0.552*	*0.115*	*0.228*
**Marital status**	Single	7.6 ± 3	7.8 ± 3	7.1 ± 3	7 ± 3	6.7 ± 3	36.2 ± 13
	Divorced	6.6 ± 3	7.2 ± 3	6 ± 4	6.4 ± 4	6.3 ± 4	32.5 ± 17
	Widow	8 ± 2	8.6 ± 2	8.2 ± 2	8 ± 3	7 ± 3	40 ± 8
	Married	7.4 ± 3	8 ± 2	7.7 ± 3	7.6 ± 3	6.5 ± 3	37 ± 12
	*P-value*	*0.285*	*0.744*	*0.062*	*0.296*	*0.982*	*0.791*
**Highest educational level**	Primary school	6.8 ± 2	8.5 ± 2	8.9 ± 2	8.4 ± 2	7 ± 3	39.8 ± 9
	Preparatory school	7.8 ± 3	8.7 ± 2	8 ± 3	7.6 ± 3	7.4 ± 3	39.7 ± 12
	High school	7.4 ± 3	7.8 ± 3	7.9 ± 3	7.7 ± 3	6.7 ± 3	36.4 ± 13
	Diploma	7.3 ± 3	8 ± 3	7.4 ± 3	7.4 ± 3	6.6 ± 3	36.7 ± 13
	College	7.6 ± 3	7.5 ± 3	7.6 ± 3	7.3 ± 3	6 ± 4	36.2 ± 13
	Post-graduate studies	7.4 ± 3	8 ± 3	7.5 ± 3	7.4 ± 3	6.6 ± 3	36.9 ± 12
	*P-value*	*0.840*	*0.487*	*0.553*	*0.922*	*0.77*	*0.904*
**Occupation**	Student	7.5 ± 3	8 ± 2	8 ± 3	7.9 ± 3	6.9 ± 3	38.3 ± 11
	Employee	7.6 ± 3	8 ± 3	7 ± 3	7.3 ± 3	6.8 ± 3	37 ± 14
	Retired	7.2 ± 3	7.8 ± 3	7 ± 3	6.8 ± 3	6 ± 4	35 ± 13
	*P-value*	*0.434*	*0.548*	0.008*	0.005*	0.049*	0.045*

*P-value is statistically significant.

On the other hand, there were no statistically significant differences observed between type I and type II diabetic patients across all the moments and total scores (P-value >0.05). Participants adhering to a regimen had higher scores in moment one: starting a medication, moment two: taking medications, moment four: reviewing medications, moment five: stopping a medication, and total scores (P-value <0.05). Participants taking oral hypoglycaemic medications had higher scores in moment three: adding a medication and moment four: reviewing medications (P-value <0.05).

Participants using insulin injections had higher scores in moment two: taking medications and adding moment three: adding a medication (P-value <0.05). Moreover, participants with controlled HbA1c had higher scores across all five moments as well as the total (P-value <0.05). No significant differences were observed between participants with or without co-morbidities, as well as for the type of healthcare provider, in any moment or total scores (P-value >0.05), “Table 5”.

**Table 5:** Associations between clinical data and medication safety practices among the studied participants (n = 473).

Clinical data		Starting a medication	Taking my medication	Adding a medication	Reviewing my medication	Stopping my medication	Total
**Type of diabetes mellitus**	Type I	7.4 ± 3	8 ± 3	7.4 ± 3	7.3 ± 3	6.8 ± 3	36.9 ± 13
	Type II	7.4 ± 3	7.9 ± 2	7.5 ± 3	7.5 ± 3	6.4 ± 3	36.8 ± 12
	*P-value*	*0.961*	*0.254*	*0.713*	*0.854*	*0.162*	*0.564*
**Following a specific regimen (diet or exercise)**	Yes	8 ± 3	8 ± 3	7.7 ± 3	7 ± 3	6 ± 3	34.5 ± 13
	No	6.6 ± 3	7.4 ± 3	7.2 ± 3	7.7 ± 3	7 ± 3	38.6 ± 12
	*P-value*	0.0001*	0.001*	*0.239*	0.018*	0.011*	0.0001*
**Taking oral hypoglycaemic medication**	Yes	7.4 ± 3	8 ± 2	7.8 ± 3	7.7 ± 3	6.7 ± 3	38 ± 12
	No	7.3 ± 3	7.7 ± 3	6.8 ± 4	6.8 ± 3	6.3 ± 3	35 ± 14
	*P-value*	*0.857*	*0.356*	0.021*	0.009*	*0.429*	*0.138*
**Taking insulin injections**	Yes	7.8 ± 3	8 ± 2	8 ± 3	7.4 ± 3	6.5 ± 3	38 ± 11
	No	7.2 ± 3	7.8 ± 3	7.3 ± 3	7.4 ± 3	6.6 ± 3	36.3 ± 13
	*P-value*	*0.051*	0.017*	0.033*	*0.844*	*0.776*	*0.193*
**Glycaemic control based on the latest HbA1c**	Controlled	7.7 ± 3	8 ± 2	7.9 ± 3	7.8 ± 3	7 ± 3	38.6 ± 11
	Not controlled	6.6 ± 3	7.4 ± 3	6.6 ± 4	6.4 ± 3	5.6 ± 3	32.7 ± 14
	*P-value*	0.0001*	0.009*	0.0001*	0.0001*	0.0001*	0.0001*
**Co-morbidities**	Present	7.3 ± 3	7.9 ± 3	7.6 ± 3	7.6 ± 3	6.4 ± 3	36.7 ± 12
	Absent	7.5 ± 3	8 ± 3	7.4 ± 3	7.3 ± 3	6.7 ± 3	37 ± 13
	*P-value*	*0.328*	*0.286*	*0.323*	*0.599*	*0.203*	*0.568*
**Type of healthcare provider**	Private sector	7.6 ± 3	8.1 ± 2	7.7 ± 3	7.5 ± 3	6.7 ± 3	37.7 ± 11
	Public sector	7 ± 3	7.8 ± 3	7 ± 4	7.2 ± 3	6.4 ± 4	35.6 ± 14
	Both	7.6 ± 3	7.8 ± 3	7.8 ± 3	7.5 ± 3	6.6 ± 3	37.3 ± 12
	*P-value*	*0.365*	*0.74*	*0.248*	*0.792*	*0.856*	*0.751*

*P-value is statistically significant.

Multiple linear regression analysis was conducted to examine factors associated with total medication safety scores. Among demographic variables, being married was a positive predictor (B = 1.331, P-value <0.05), and being retired was a negative predictor (B = −2.092, P-value <0.05). Regarding clinical characteristics, several factors were positively associated with higher medication management scores: following a specific regimen (diet or exercise) (B = 3.052, P-value <0.05), taking oral hypoglycaemic medication (B = 3.079, P-value <0.05), taking insulin injections (B = 3.837, P-value <0.05), and glycaemic control based on the latest HbA1c (B = 5.673, P-value <0.05), “Table 6”.

**Table 6:** The multivariate regression analysis of medication safety practices among the studied participants (n = 473).

	Unstandardized Coefficients		Standardized Coefficients	t	P-value	95.0% Confidence Interval for B	
	B	Std. Error	Beta			Lower Bound	Upper Bound
Age	−0.077	.057	−.100	−1.348	.178	−.189	.035
Sex	0.824	1.218	.033	.676	.499	−1.569	3.217
Marital status	1.331	.623	.137	2.137	.033*	.107	2.555
Highest educational level	−.293	.499	−.027	−.587	.558	−1.272	.687
Occupation	−2.092	.722	−.154	−2.898	.004*	−3.511	−.674
Type of diabetes mellitus	−.293	1.202	−.012	−.244	.807	−2.656	2.070
Following a specific regimen (diet or exercise)	3.052	1.135	.122	2.689	.007*	.821	5.284
Taking oral hypoglycaemic medication	3.079	1.395	.113	2.207	.028*	.337	5.822
Taking insulin injections	3.837	1.271	.145	3.018	.003*	1.338	6.335
Glycaemic control based on the latest HbA1c	5.673	1.247	.208	4.548	.0001*	3.222	8.124
Co-morbidities	−1.801	1.178	−.073	−1.529	.127	−4.116	.514
Type of healthcare provider	−.421	.719	−.026	−.586	.558	−1.833	.991

*P-value is statistically significant.

## Discussion

This study demonstrated that diabetic patients have high adherence to the WHO “Five Moments for Medication Safety,” with the highest scores in Moment Two (“taking medications”) and the lowest in Moment Five (“stopping medications”), consistent with previous studies in Saudi Arabia [19–21]. This finding suggests that patients are actively engaged in safe medication use, but they may undervalue some risks associated with medication discontinuation and cessation practices.

Moment Two emphasizes taking the right medication, dose, route, time, and technique, and diabetic patients in the study generally perform well in these tasks due to daily disease management needs. For example, diabetic patients on oral hypoglycaemic agents often follow pre-set dosing schedules reinforced by healthcare providers [22], while insulin users receive regular instruction on proper storage and injection [19]. Other factors like immediate physiological consequences of missed or incorrect doses, coupled with reinforcement during clinic visits, education programs, and family support, sustain high adherence [23].

In contrast, Moment Five (“stopping medications”) scored the lowest. This domain involves items like proper discontinuation schedules, reporting of side effect, medication refills, and disposal. Lower adherence among diabetic patients in this study may reflect patients’ underestimation of the risks of stopping or disposing medications incorrectly. Many patients perceive stopping medication as safe and underestimate potential harm, such as uncontrolled hyperglycemia or environmental hazards from improper disposal [24–26]. Limited education focusing on the “end-of-use” phase further contributes to weaker practices in this domain, as healthcare providers typically emphasize initiation and administration over discontinuation [27,28].

Clinical factors including adherence to a regimen, use of oral hypoglycaemic agents or insulin, and controlled HbA1c, were positively associated with higher medication safety scores. This likely reflects the greater disease awareness and self-management among these diabetic patients. The adherence of diabetic patients to diet or exercise plans may be higher when they have higher health literacy and self-control, which in turn extend to safe medication practices [29].

Frequent healthcare visits for patients on oral hypoglycaemic agents or insulin reinforce their adherence to medication safety rules [30]. Similarly, controlled HbA1c suggests effective medication regimens and an understanding of treatment precision [31]. These findings underscore the importance of proactive self-management and ongoing patient-provider communication to enhance medication safety in diabetes care.

## Limitations

Despite the valuable insights provided by this study, it has several limitations. First, the cross-sectional design can’t confirm the causal relationships between clinical or demographic variables and medication safety practices. Second, data were gathered via a self-administered questionnaire, which depends on the own understandability of the participants and may be influenced by recall and social desirability bias, causing participants to overrate or underestimate their safety practices. Additionally, the sampling technique, which was non-random, adds to the restriction of the generalizability of findings; however, the relatively large sample size could reduce this risk.

## Conclusion

The study reported that diabetic patients generally showed a high adherence to the “Five Moments for Medication Safety,” with the highest practice observed in moment two: “taking medications” and the lowest in moment five: “stopping medications”. Clinical factors, including adherence to a regimen, use of oral hypoglycaemic agents or insulin, and controlled HbA1c, were significantly associated with higher medication safety practices.

## Recommendations

These findings highlight the importance of patient engagement in promoting safe medication practices among diabetic patients. As a result, educational initiatives should be implemented to emphasize the crucial role of patients in promoting overall patient safety, particularly in medication safety. Considering the patient as an active member in the healthcare team, they should share in the healthcare decisions, particularly when related to their medication. Integrating the “WHO Five Moments for Medication Safety” into the curricula of medical, pharmacological and nursing students can improve their understanding of safe medication practices and promote a culture of patient safety from early training. Further research should be conducted among other chronic diseases’ patients and those with polypharmacy as well. Also, research to assess these moments from the healthcare providers’ perspective should be conducted to capture the full picture of medication safety.

## Supporting information

S1 Appendix The questionnaire used in this study is provided. (DOCX)

S1 Data The raw data underlying this study can be found in the supporting Excel file. (XLSX)
