# Supplementary material for: WHO five moments for medication safety among diabetic patients in Saudi Arabia
Source: PLoS One. 2026 Apr 10;21(4):e0346935. doi: 10.1371/journal.pone.0346935 (PMC13068258; doi:10.1371/journal.pone.0346935)
Supplement: S1 Appendix — (DOCX) [file pone.0346935.s001.docx]

**S1 Appendix**

**Questionnaire**

**Section (1): General characteristics**

1. **What is your age (in years)?** ……………………….
2. **What is your sex?** m/f
3. **What is your marital status?**

- Single
- Divorced
- Widowed
- Married

1. **What is your educational level?**

- Primary education
- Intermediate education
- Secondary education
- Diploma
- Bachelor’s degree
- Postgraduate studies

1. **What is your employment status?**

- Retired
- Student
- Employed

**Section (2): Clinical data**

1. **Type of diabetes**

- Type 1 diabetes
- Type 2 diabetes

1. **Do you follow a specific regimen (such as a diet or exercise) to manage diabetes?**

- Yes
- No

1. **Do you take oral hypoglycemic medications?**

- Yes
- No

1. **Do you take insulin injections?**

- Yes
- No

1. **According to your last HbA1c test, is your blood sugar well controlled?**

- Yes
- No

1. **Do you have other chronic diseases?**

- Yes
- No

1. **Your healthcare provider(s):**

- Private sector
- Government sector
- Both

**Section (3):**

**Please select the items you frequently do when contacting your healthcare provider about your medications:**

| **Starting a medication** | **Never** | **Sometimes** | **Always** |
| --- | --- | --- | --- |
| 1. What is the name of this medication and what is it for? |  |  |  |
| 1. What are the risks and possible side-effects? |  |  |  |
| 1. Is there another way of treating my condition? |  |  |  |
| 1. Have I told my health professional about my allergies and other health conditions? |  |  |  |
| 1. How should I store this medication? |  |  |  |
| **Taking my medication** |  |  |  |
| 1. When should I take this medication and how much should I take each time? |  |  |  |
| 1. How should I take the medication? |  |  |  |
| 1. Is there anything related to food and drink that I should know while taking this medication? |  |  |  |
| 1. What should I do if I miss a dose of this medication? |  |  |  |
| 1. What should I do if I have side-effects? |  |  |  |
| **Adding a medication** |  |  |  |
| 1. Do I really need any other medication? |  |  |  |
| 1. Have I told my health professional about the medications I am already taking? |  |  |  |
| 1. Can this medication interact with my other medications? |  |  |  |
| 1. What should I do if I suspect an interaction? |  |  |  |
| 1. Will I be able to manage multiple medications correctly? |  |  |  |
| **Reviewing my medication** |  |  |  |
| 1. Do I keep a list of all my medications? |  |  |  |
| 1. How long should I take each medication? |  |  |  |
| 1. Am I taking any medications I no longer need? |  |  |  |
| 1. Does a health professional check my medications regularly? |  |  |  |
| 1. How often should my medications be reviewed? |  |  |  |
| **Stopping my medication** |  |  |  |
| 1. When should I stop each medication? |  |  |  |
| 1. Should any of my medications not be stopped suddenly? |  |  |  |
| 1. What should I do if I run out of medication? |  |  |  |
| 1. If I have to stop my medication due to an unwanted effect, where should I report this? |  |  |  |
| 1. What should I do with leftover or expired medications? |  |  |  |
